# Does Global and Local Vision Have an Impact on Creative and Analytic Thought? Two Failed Replications

**DOI:** 10.1371/journal.pone.0132885

**Published:** 2015-10-15

**Authors:** Karl Christoph Klauer, Henrik Singmann

**Affiliations:** 1 Institut für Psychologie, Albert-Ludwigs-Universität Freiburg, Freiburg, Germany; 2 Psychologisches Institut, Universität Zürich, Zürich, Switzerland; Goldsmiths, University of London, UNITED KINGDOM

## Abstract

According to GLOMO^sys^ (the GLObal versus LOcal processing MOdel, a systems account), an important distinction is that between a local and a global processing system: The former processes information in parts, the latter processes it globally. These systems can be activated by perceptual processing and carry over to subsequent conceptual processing, in particular to analytical and creative thought. A conceptual and a high-powered close replication of previously reported studies test predictions of GLOMO^sys^ for analytical thought and for analytical and creative thought, respectively. The present studies found no evidence that processing style primed via the Navon letter task has an impact on creative or analytic thought.

## Introduction

According to Förster and Danneberg’s GLOMO^sys^ [1] (see also [2]), an important distinction is that between a local and a global processing system: The former processes information in parts, the latter processes it globally. These systems can be activated by perceptual processing and carry over to subsequent conceptual processing.

**Fig 1:**
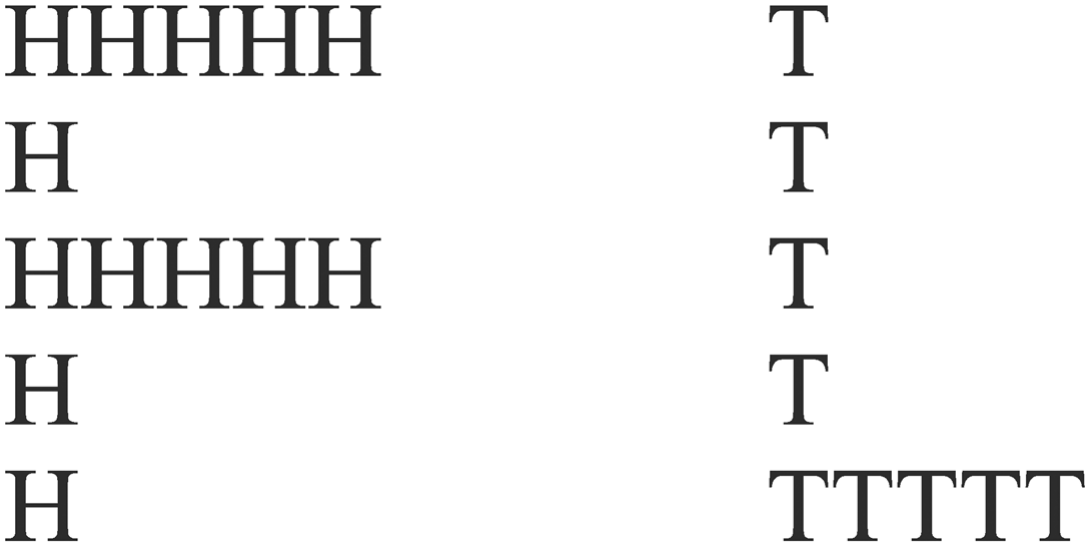
Two Navon letters. To the left: The large letter F made up of small letters H. To the right: The large letter L made up of small letters T.

Navon’s letter task [3] is often used to induce either local or global perceptual processing. The task involves figures of large letters made up of many instances of a specific small letter (e.g., the large letter F made up of many small letters H; see Fig 1). In the version used to induce a processing style (e.g., [4]), two letters (L and H) are mapped on two response keys, and participants are to press the key corresponding to the letter which is seen in the Navon figure. To induce local (global) processing, figures are shown that always employ one of the target letters L and H as small (large) letter; for a control condition, target letters occur as small or large letter in equal proportion.

Regarding subsequent conceptual processing, the focus here is on analytical and creative processing. The local processing system supports analytical processing as required in syllogistic reasoning tasks; the global system creative processing [1, 2]. For example, global perceptual processing leads to broad conceptual attention disposing people to activate superordinate concepts. Furthermore, “activation of superordinate concepts gives room for further association of remote associates … and thereby should support creative generation … In contrast, many analytical tasks (such as syllogisms of the sort ‘If A < B and C > B, then ?’) profit from a narrow and systematic focus on the information given and use of deductive reasoning to draw conclusions from that information” [2].

The present work is motivated by studies reported by Förster and Denzler [5] (retraction: [6]) that focused on testing the above prediction of GLOMO^sys^ for creative and analytical tasks. Prior to the publication of the events that eventually led to the retraction, we became aware of this work, henceforth referred to as FD. The large and robust effects reported therein encouraged us to attempt a conceptual replication probing for an effect of perceptual processing style on a classical analytical reasoning task, conditional syllogisms, reported as Study 1 here. An effect of local-global processing style on syllogistic reasoning would be interesting in light of dual-process and dual-system accounts in the thinking and reasoning literature [7] that might be aligned with a local-global distinction.

After doubts had been raised regarding FD and two other papers, we joined an international group, headed by Klaus Fiedler and Nira Liberman, who invited attempts to replicate experiments reported in these three papers to (re-)establish the validity of the Förster et al. results in a joint validation program. We decided to replicate Experiment 6 reported by FD. The replication is reported as Study 2 below. It examines the above-discussed GLOMO^sys^ predictions for both analytical and creative processing. The data, stimuli, materials, experimental programs, analyses scripts, and additional analyses for both studies can be accessed as an online supplement posted on the open science framework under the URL: https://osf.io/ya5w9/. Outside the just-mentioned replication initiative, an independent replication of Experiment 1 by FD is also being conducted [8].

## Study 1

Across five experiments, involving seven replications with between 45 and 60 participants, FD reported significant effects of processing style on an analytical reasoning task with smallest effect size across replications of $\hat{f}=0.50$. In Study 1, we implemented a sample size *n* of 75 implying a test power of .97 for detecting a main effect of processing style of this magnitude. The Navon letter task was used to induce different processing styles, the dependent variable was performance in a standard conditional reasoning task.

### Methods

#### Ethics Statement

The ethical principles as formulated in the WMA Declaration of Helsinki guided our research project. If research objectives do not involve issues regulated by law (e.g., the German Medicine Act [Arzneimittelgesetz, AMG], the Medical Devices Act [Medizinproduktegesetz, MGP], the Stem Cell Research Act [Stammzellenforschungsgesetz, StFG] or the Medical Association’s Professional Code of Conduct [Berufsordnung der Ärzte]), then no ethics approval is required for social science research in Germany. Our studies have no such objectives, and therefore, no IRB approval or waiver of permission was sought for these studies.

Participants were recruited from the participant pool of the first author’s department that consists of persons who have given written permission to receive invitations via e-mail to participate in psychological experiments at that department. Participation in both studies was entirely voluntary. Participants were informed of the study procedures, of their anonymity, and they were told that they were free to leave the study at any point if they so wished. Participants provided written consent on the basis of this information prior to participating.

These procedures were in accordance with the German Society for Psychology’s research standards (Grundsätze der Forschung am Menschen, C.III, para. 6).

#### Participants

Participants were mostly University-of-Freiburg students with different majors. They received 3.50 Euro as gratification for participating. There were 75 participants, one of whom decided not to complete the experiment, after having worked on only two conditional problems. Of the remaining 74 participants, 29 were male, 44 female, and one participant chose not to respond to the question regarding his or her sex. Mean age was 23.3 years (*SD* = 3.2). Participants were randomly assigned to one of the three processing-style groups with 25 participants each in the local and control group, and 24 in the global group.

#### Navon letter task

We used a standard Navon letter task [4] in the same version as per FD’s report, whose description we therefore reproduce almost verbatim: Participants worked on the computerized Navon letter task in which a series of global letters (6.5 × 9.5 cm) made up of local letters (0.9 × 1.1 cm) are presented. Upon presentation of a fixation cross (“+”) in the center of the screen for 500 ms, 1 of 8 global composite letters (e.g., an “H” made of “F”s; an “L” made of “H”s; an “F” made of “H”s; an “F” made of “L”s) was randomly presented, and participants were instructed to press one response key if the stimulus contained the letter “L” and to press a different response key if the stimulus contained the letter “H”. Participants were asked to respond as quickly as possible. In case of an error, the word “FEHLER” [ERROR] was shown for 1.5 seconds. For the global (local) priming condition, Ls and Hs were always the global (local) letter (48 trials); in the control condition, targets occurred as both, small and large letters (24 local and 24 global trials).

#### Conditional reasoning task

The conditional syllogisms consist of (a) a major premise, which is a conditional statement in the form “if p, then q”, (b) a minor premise, which is one of the statements p, q, or one of their negations, and (c) a conclusion. For example, one problem was: “If a person has fallen into a swimming pool, then the person is wet. A person has fallen into a swimming pool. Conclusion: The person is wet.” Participants’ task was to judge in a binary “yes” versus “no” format whether the conclusion was logically valid, that is whether it followed necessarily from the premises. Participants were asked to ignore their background knowledge and to consider the conditional statement true (“one hundred percent and without exception”) even if background knowledge suggested otherwise (discounting, for example, the possibility that the swimming pool is empty) and to consider the minor premise a given fact. They were also reminded of the fact that the statement “If a, then b” is not the same as the statement “If b, then a” to forestall a relatively frequent bidirectional interpretation of the conditional statement.

There were nine conditional statements that served as major premise. For each conditional, we presented four classical syllogisms that differ in minor premise and conclusion, for a total of 36 = 9(conditionals) × 4(syllogisms) problems. The four classical syllogisms are modus ponens (MP: minor premise p; conclusion q; see the above example), affirmation of the consequent (AC: minor premise q; conclusion p), denial of the antecedent (DA: minor premise not-p; conclusion not-q), and modus tollens (MT: minor premise not-q, conclusion not-p). Under a traditional logical interpretation of the conditional, MP and MT are logically valid inferences, whereas AC and DA are not logically valid.

The nine conditional statements were the same as used by [9] (Exp. 2). Based on normative data from previous studies, three were “prological” in the sense that world knowledge suggests that p is sufficient for q, but not as much q for p, thereby supporting the acceptance of the logically valid MP and MT conclusions, albeit on extra-logical grounds. The above example is one of the used prological conditionals. Three more were “counterlogical” in the sense that world knowledge suggests that q is sufficient for p, but not as much p for q (e.g., If you water a plant well, then the plant stays green) supporting the acceptance of the logically invalid AC and DA problems; three were neutral in the sense that there was equal support (or equal lack of support) for both the sufficiency of p for q and the sufficiency of q for p in the normative data (e.g., “If a person studies hard, then the person will get a good grade in the test”).

The 36 resulting conditional syllogisms were presented in random order with the 4 conditional syllogisms per conditional statement following each other immediately in random order. Working through the 36 problems required less than 10 min.

#### Procedure and Design

The experiment was run on personal computers in individual sessions. Participants were told that they were to work on two short and unrelated tasks combined in one session. They first worked through the Navon letter task, followed by the conditional reasoning task. The session ended with the question whether the participants had the feeling that the two tasks had an influence on each other, followed by a demographic questionnaire. In case of a “yes” response to the influence question, participants were asked to explain the nature of the influence. There were only two “yes” responses. The two participants’ explanations of the nature of the influence can be found in the online supplement under the above URL. The study implements a design with between-participants factor processing style (local, control, global) and within-participants factors type of syllogism (counterlogical, neutral, prological) and inference (MP, MT, AC, and DA).

#### GLOMO^sys^ Predictions

According to GLOMO^sys^, performance in the conditional reasoning task should be highest in the local processing-style condition, intermediate in the control condition, and lowest in the global processing-style condition.

### Results and Discussion

**Fig 2:**
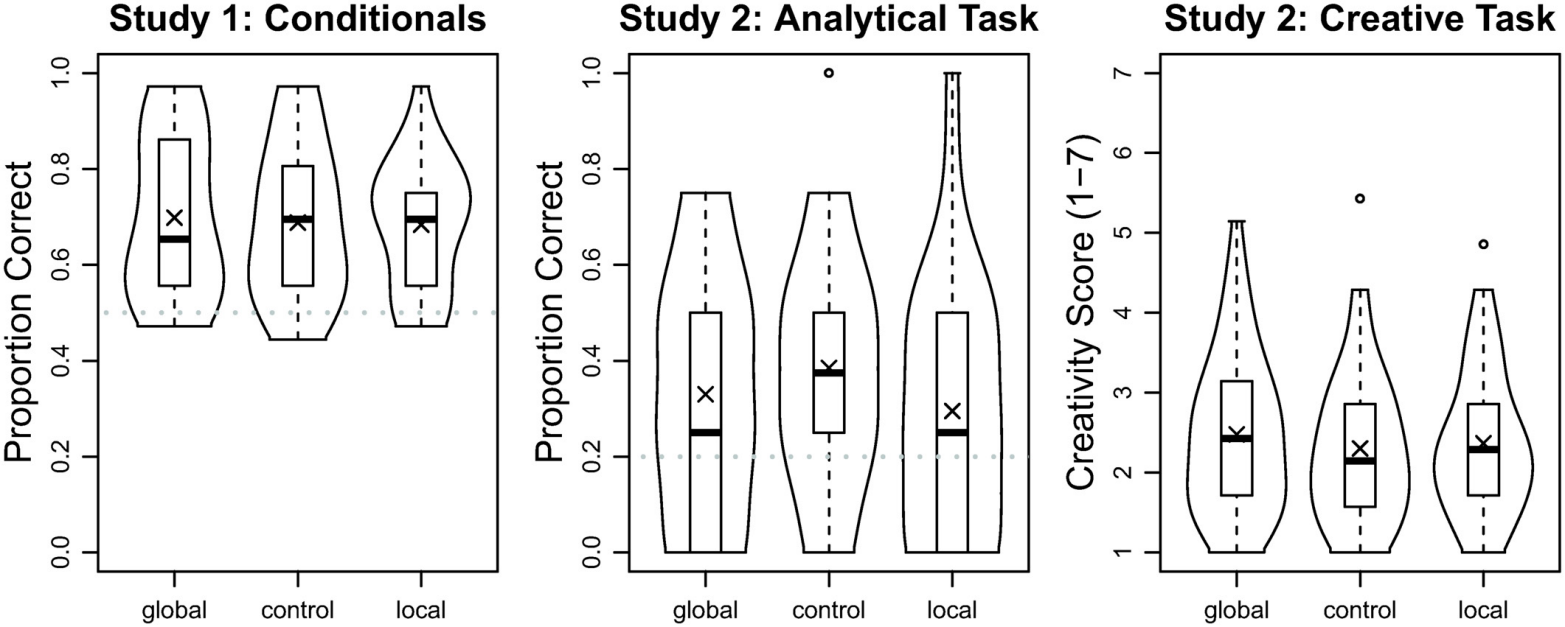
Violin plots of the data for Studies 1 and 2. The boxes give the interquartile range; the crosses the means; the bold horizontal lines in the boxes the medians; the dotted grey lines chance performance for the analytical tasks; overlaid to the left and the right are kernel density estimates of the distribution of the data.

Mean accuracy in the local, control, and global processing-style condition was 68% (*SD* = 13), 69% (*SD* = 16), and 70% (*SD* = 17), respectively; see Fig 2 for more information. An analysis of variance with between-participants factor processing style and within-participants factors type of syllogism and inference revealed no significant effects or interactions for processing style (largest *F* = 0.87, smallest *p* = .49). Qualitatively identical results were obtained for logit regression reported in the online supplement (see URL above) along with the full analysis-of-variance results and additional analyses. Cohen’s d for the contrast of local versus global processing-style conditions was *d* = −0.10.

The test power for detecting effects as strong as those reported by FD ($\hat{f}\geq 0.50$) was large (1 − *β* ≥ .97) so that we can be confident that an effect this large would have been detected. Observed effect sizes are, however, likely to overestimate true effect sizes due to publication bias ([10, 11]). In terms of Cohen’s classification of effect sizes, *f* = 0.40 is a strong effect, *f* = 0.25 an effect of medium size, and *f* = 0.10 of small size [12]. Test power 1 − *β* for effects of these sizes are, respectively, .86, .45, and .11. The present study has 80% power to detect an effect of size *f* = 0.37; the power of FD’s studies to detect effects of this size is 1 − *β* = .69.

Furthermore, the Bayes factor (Bf) evidence for the null hypothesis in a one-tailed *t* test comparing global and local condition [13, 14] amounted to 4.39 (conventionally classified as “substantial”, e.g. [15]) and in the ANOVA design with factor condition (local, neutral, global) to 2.94. Just as classical power analyses depend strongly on the assumed effect sizes, the outcome of Bf analyses depends strongly on the mean and spread of the assumed prior effect-size distribution, and we used the recommended distributions [13, 14] (see the online supplement under the URL above for details). For example, given the present settings, the largest *t* value that produces substantial Bf evidence for the null hypothesis (i.e. BF ≥ 3.0) amounts to 0.21 [16] and is associated with an effect size estimate of *d* = 0.06.

Taken together, there was little evidence for an effect of, or an interaction involving, processing style. We were of course disappointed by this result, but did not want to make much of it, given the well-known problems with interpreting failed replications some of which are reviewed in the General Discussion.

For example, although we used the same Navon letter task to induce processing styles as previous work, the analytical task differed from the one employed by FD. Hence, it could be argued that something about the particular instantiation of analytical thought used here prevented the effect from emerging. For example, it may be the case that the present reasoning task is for some unknown reason more or less insensitive to manipulations of processing style in contrast to most other analytical tasks one might have chosen. Given that the conditional-syllogisms task is, however, sensitive to manipulations of related processing modes (e.g., [9]) such as probabilistic and deductive processing and given that these processing modes can be roughly aligned with the distinction between a global and a local processing style, this is perhaps an unlikely possibility, but it cannot be ruled out.

## Study 2

Study 2 was designed as a close replication of FD’s Experiment 6. This involved an analytical task and a creative task as already employed by [17] and [18], respectively. We used copies of the stimuli and materials provided by Jens Förster. Following a recommendation by [19] for close replications, we used 2.5 times the sample size employed in the original study and thus, an *n* of 50 per processing-style group.

### Methods

The Navon letter task was the same as already described with the following changes intended to comply more closely with the details on FD’s original procedures that were made available to members of the international replication initiative. Thus, the instructions were slightly changed in that we now used the original instructions by FD as reproduced in the online supplement (see URL above). In addition, there was no error feedback in the Navon task in the case of wrong responses. Finally, the size of a global letter was now 2.1 cm horizontally and 2.7 cm vertically; that of a local letter was 0.5 × 0.5 cm.

#### Analytical task

The analytical task was described by [17] as follows: “Participants worked on four logic problems from the analytical reasoning section of the GRE, translated into German. These problems involve evaluating the truth value of a number of propositions given an initial set of basic facts. Unlike insight-related tasks, the current problems demand little in the way of mental restructuring and do not require cognitive search for information beyond that provided. Rather, solving these problems involves systematically organizing the information given and carefully analyzing it to determine the verity of a series of logical conclusions”.

Like FD, we instructed participants that they had 4 min to solve the tasks. Participants were provided paper and pencil in line with the task instructions on the GRE questionnaire stating that it may be helpful to draw a rough diagram. After 4 min, a 30 s countdown appeared on the computer screen, and participants were informed that their responses would be automatically registered at the end of the countdown.

#### Creative task

For the creative task, participants saw a cartoon picture of a dog sitting on a sofa. Their task was to find the most creative title for it. They had 2 min to finish the task. Again, we provided a 30 s countdown after 2 min had elapsed, and participants were informed that their responses would be automatically registered at the end of the countdown. Seven judges who were blind to the conditions rated the creativity on a 7-point scale (1 = *not creative at all* to 7 = *very creative*). Interrater reliability was .81 in terms of Cronbach’s alpha and thus a bit lower than that reported by FD (*α* = .85), but a bit higher than that reported by [17] (*α* = .76). Ratings were averaged across raters for the measure of creativity.

#### Participants

The same Ethics statement applies as for Study 1. Participants were mostly University-of-Freiburg students with different majors. They received 3.50 Euro as gratification for participating. There were 158 participants. Eight were excluded from the analyses, because the experimenters forgot to provide paper and pencil for the analytical task. These were replaced by new participants so as to ensure 50 participants each in the local, control, and global group. For four of the 150 participants retained for the analyses, demographic data were lost, due to computer crashes in administering the final demographic questionnaire. Of the 146 participants for whom demographic data are available, 67 were male, 77 female, and 2 chose not to respond to the question regarding their sex. Mean age was 22.9 years (*SD* = 3.9).

#### Procedure and Design

The experiment was run on personal computers in individual sessions. Participants first worked through the Navon letter task, followed by the analytical and the creative task. The order of these two tasks was counterbalanced across participants in each processing-style condition. The session ended with the question whether the participants had the feeling that the two tasks had an influence on each other, followed by a demographic questionnaire. In case of a “yes” response to the influence question, participants were asked to explain the nature of the influence. The participants’ responses can be found in the online supplement (see the URL above).

The study implements a design with between-participants factor processing style (local, control, global). There are two dependent variables, performance in the analytical task and performance in the creative task.

#### GLOMO^sys^ Predictions

According to GLOMO^sys^, performance in the analytical task should be highest in the local processing-style condition, intermediate in the control condition, and lowest in the global processing-style condition. Conversely, performance in the creative task should be lowest in the local processing-style condition, intermediate in the control condition, and highest in the global processing-style condition.

### Results and Discussion

#### Analytical task

Mean performance in the local, control, and global processing-style condition in terms of number of items correctly responded to was 1.18 (*SD* = 1.06), 1.54 (*SD* = 1.03), and 1.32 (*SD* = 1.02), respectively; see Fig 1 for more information. An analysis of variance with between-participants factor processing style revealed no significant effect of processing style: *F*(2, 147) = 1.53, *p* = .22, $\hat{f}=0.08$. Qualitatively identical results were obtained for logit regression reported in the online supplement (see URL above). Cohen’s d for the contrast of local versus global processing-style conditions was *d* = −0.13.

The test power for detecting effects as strong as those reported by FD ($\hat{f}\geq 0.50$) was large (1 − *β* ≥ .9999) so that we can be confident that an effect this large would have been detected. This is also true for a strong effect according to [12]: *f* = 0.40, 1 − *β* = .99; for an effect of medium size: *f* = 0.25, 1 − *β* = .78; for a small effect: *f* = 0.10, 1 − *β* = .18. The present study has 80% power to detect an effect of size *f* = 0.26; the power of FD’s studies to detect effects of this size is 1 − *β* = .39.

Furthermore, the Bf evidence for the null hypothesis in a one-tailed *t* test comparing global and local condition amounted to 7.35, counting again as substantial, and in the ANOVA design with factor condition (local, neutral, global) to 1.92. Given the present settings, the largest *t* value that produces substantial Bf evidence for the null hypothesis (i.e. BF ≥ 3.0) amounts to 0.54 and is associated with an effect size estimate of *d* = 0.11.

Following [19], the effect size *d*_33%_ that gives the original experiment a power of 1/3 is 0.50. Ihe effect size of the local/global contrast (*d* = −0.13) is significantly smaller than *d*_33%_, *p* < .001 (see [19]).

#### Creative task

Mean rated creativity in the local, control, and global processing-style condition was 2.37 (*SD* = 0.91), 2.30 (*SD* = 0.96), and 2.48 (*SD* = 0.99), respectively; see Fig 1 for more information. An analysis of variance with between-participants factor processing style revealed no significant effect of processing style: *F*(2, 147) = 0.44, *p* = .64, $\hat{f}=0.00$. Cohen’s d for the contrast of local versus global processing-style conditions was *d* = 0.12.

Across seven replications, the smallest effect size for an effect on creative thought reported by FD was $\hat{f}=0.49$. Test power for detecting an effect of this size was 0.9998 so that we can be confident that we would have detected a real effect of this size. Test power for detecting small, medium-sized, and strong effects is as just reported for the analytical task.

The Bayes factor evidence for the null hypothesis in a one-tailed *t* test comparing only global and local condition amounted to 2.87 and in the ANOVA design with factor condition (local, neutral, global) to 3.23, counting again as substantial. Again, the effect size of the local/global contrast (*d* = 0.12) is significantly smaller than *d*_33%_ = 0.50, *p* = .03.

#### Discussion

The relatively close replication of FD’s Experiment 6 was not successful. Despite a sample size 2.5 times as large as the one reported by FD [19], and high test power for detecting effects of the sizes originally reported, we found neither evidence for an effect of processing style on analytical nor on creative thought.

With hindsight, it is perhaps plausible that strong results could not be expected. In our assessment and in participants’ performance, the analytical reasoning task is quite difficult, restricting the possibility for differences between conditions to emerge due to floor effects. The creativity measure is based on only one item, the analytical task on only four dichotomous items, suggesting that neither measure may be sufficiently reliable to permit strong effects to emerge against the probably substantial background of noise and of pre-existing individual differences in analytical and creative skills.

## General Discussion

The discussion can be short. We presented a conceptual and a close replication of results reported by FD. The sample size for the close replication followed recommendations to implement 2.5 times the original sample size [19]. As already mentioned, it was preregistered as part of an international initiative to replicate results from papers by Jens Förster that have come under critique. The sample size for the conceptual replication was smaller, but large enough to detect effects of the size reported by FD.

The present studies share a couple of generic limitations of failed replications. For example, Jens Förster has recommended a couple of measures regarding the clothing and demeanor of the experimenters that he considers conducive to the effects (see online supplement under the URL above). Although we heeded most of these, it is possible that unknown factors of this kind prevented a true effect from generalizing to our setting.

Furthermore, as pointed out by Klaus Fiedler in an unpublished manuscript, in testing a prediction of the form “If X, then Y”, it has to be ensured that X was given and Y was validly measured. In consequence, Fiedler recommends to add validity checks for the manipulation of processing style (X) and for the dependent variable (Y). It is clear that validity checks of this kind add to the scientific value of replication attempts.

The present work addressed a logically prior question in which X and Y are understood as the operationalizations described by FD rather than as higher-level theoretical constructs. The question is: Can the reported effect be replicated when the operations and procedures as described in the original report are implemented faithfully? Many researchers have argued that close replications of this kind can fulfill an important role in general (e.g., [20]) and in the debate around some of Förster’s work in particular (e.g., [21]). For example, in one prominent theory-of-science view, a basic claim of an experimental paper is that a low-level regularity exists in the real world; namely that implementing the operations detailed in the procedures section makes the reported effect appear reliably. In this view, low-level laws of this kind are really the only “data” with theoretical import, and close replications test whether a low-level regularity of the postulated kind actually exists in the world. The insistence on replicable results establishing low-level laws rather than on singular experiments reflects the acknowledgment that in a complex multicausal world a single experimental outcome may be an artefact of an unusual and historically unique constellation of factors (e.g., [22]).
